# Riboflavin treatment in L-2-hydroxyglutaric aciduria: report on a pediatric patient and literature review

**DOI:** 10.1007/s13353-025-00974-4

**Published:** 2025-05-17

**Authors:** Patryk Lipiński, Elżbieta Ciara, Anna Bogdańska, Elżbieta Jurkiewicz, Anna Tylki-Szymańska

**Affiliations:** 1 Institute of Clinical Sciences, Maria Skłodowska-Curie Medical Academy, Plac Żelaznej Bramy 10, 00-136, Warsaw, Poland; 2 Department of Medical Genetics, Children’s Memorial Health Institute, Warsaw, Poland; 3 Department of Clinical Biochemistry, The Children’s Memorial Health Institute, Warsaw, Poland; 4 Department of Diagnostic Imaging, The Children’s Memorial Health Institute, Warsaw, Poland; 5 Department of Pediatric Nutrition and Metabolic Diseases, Children’s Memorial Health Institute, Warsaw, Poland

**Keywords:** Novel variant; L-2-hydroxyglutaric aciduria; L2HGDG gene; Riboflavin

## Abstract

L-2-hydroxyglutaric aciduria (L-2-HGA, #236,792) is an autosomal recessive neurodegenerative disorder caused by the deficiency of L-2-hydroxyglutarate dehydrogenase, a flavin adenine dinucleotide (FAD)-dependent enzyme, due to biallelic pathogenic variants in the *L2HGDH* gene. The present study described the patient with L2HGA presenting with a slight psychomotor delay, epilepsy from 5 years of age, non-progressive cerebellar ataxia, and mild to moderate intellectual disability during 10 years of follow-up. Two different heterozygous variants in the *L2HGDH* gene were identified in the patient: a known substitution c.829C > T(p.Arg277*) and a novel substitution c.1196 + 1G > A corresponding with significantly increased urinary L-2-hydroxyglutarate (L2HG) excretion. A 6-month period of treatment with riboflavin (100 mg/day) was implemented with no clinical nor biochemical effect.

## Background

L−2-hydroxyglutaric aciduria (L-2-HGA, # 236,792) is an autosomal recessive neurodegenerative disorder caused by the deficiency of L-2-hydroxyglutarate dehydrogenase, a flavin adenine dinucleotide (FAD)-dependent enzyme, due to biallelic pathogenic variants in the *L2HGDH* gene (Rzem et al. 2004, 2007; Schaftingen et al. 2009). L2HGA usually manifests in the first years of life with a slowly progressive course of cerebellar ataxia, and other neurological features including macrocephaly, epilepsy, and pyramidal and extrapyramidal signs (Bozaci et al. 2023; Fayed et al. 2024; Gunduz et al. 2022; Sass et al. 2008; Mainka et al. 2020). The biochemical hallmark of L2HGA is an increased concentration of L-2-hydroxyglutaric acid in body fluids, including urine, cerebrospinal fluid, and plasma (Rzem et al. 2007; Schaftingen et al. 2009). No specific treatment exists for L2HGA; however, several reports suggest that flavin adenine dinucleotide (FAD) sodium or its precursor—riboflavin—could be considered a potential therapeutic approach (Yilmaz 2009; Ahmed et al. 2023).

Besides of the consistent clinical, biochemical, and neuroradiological features of L2HGA, the long-term observational studies are sparse, while the potential treatment with riboflavin needs further assessment. The aim of this paper was to provide an analysis of riboflavin treatment in L2HGA based on report of pediatric patient as well as literature review.

## Case report

### Clinical and biochemical data

A 5-year-old girl was hospitalized due to generalized clonic-tonic seizures, observed for the first time in her life. The patient was the second child of healthy Polish parents, born at 38 weeks of gestation by a spontaneous delivery after an uncomplicated pregnancy, with a birth mass of 3650 g and head circumference of 36 cm. Her psychomotor development was assessed to be normal—she was able to sit independently at 6 months of age and walk independently at 14 months of age; she spoke her first words at 8 months of age.

**Fig. 1:**
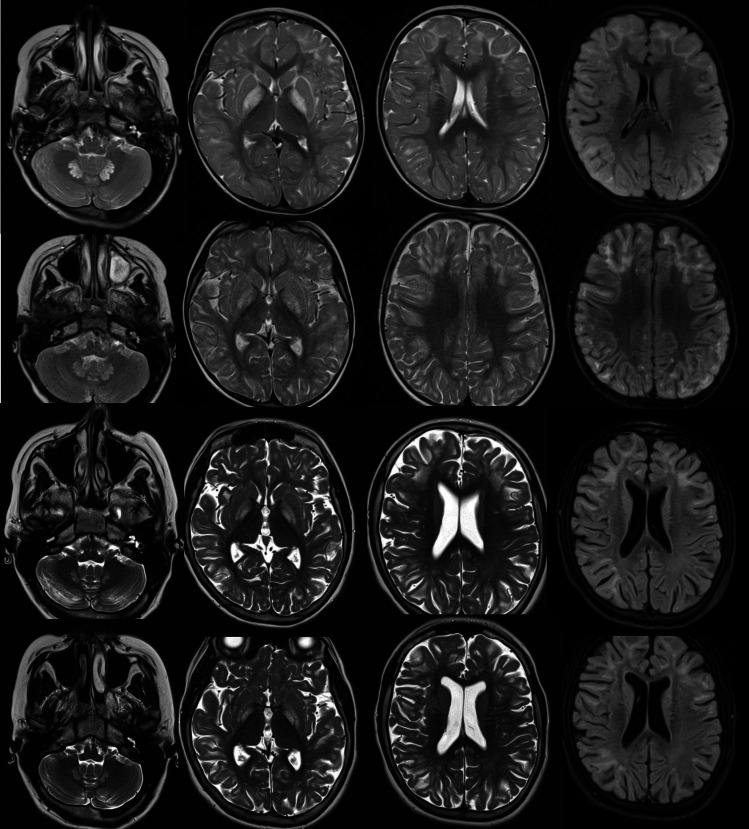
Brain MR examinations, T2 W, and FLAIR images

On admission, the patient presented with the head circumference of 51 cm (50–75 percentile), mild intellectual disability, and ataxic gait. Normal results of basic laboratory tests, including serum glucose, ammonia, lactate, and blood gases, were noted. Eye fundus examination was normal. Brain MRI demonstrated leukodystrophic changes with an increased intensity within the globi pallidi and dentate nuclei on T2-weighted images (Fig. 1). Echocardiography was normal. Psychologic tests confirmed a mild intellectual disability (based on the Leiter international performance scale). During hospitalization, several episodes of generalized tonic–clonic seizures were observed. Electroencephalogram revealed a generalized paroxysmal activity. Valproic acid was implemented for the treatment of epilepsy with a good seizure control.

Based on the clinical and MRI features, inborn errors of metabolism work-up were commenced. Gas chromatography–mass spectrometry analysis delineated significantly increased urinary L-2-hydroxyglutarate (L2HG) excretion of 258 mmol/mol creatinine. Serum amino acid analysis revealed only elevated lysine concentration (299 µmol/L; reference ranges, 66–266). Molecular analysis (*L2HGDH* gene sequencing) was performed to confirm the clinical and biochemical diagnosis of L2HGA.

Brain MRI performed 1 year later revealed the same features as earlier (Fig. 1).

During 10-year follow-up, the patient presented with epilepsy controlled with valproic acid, non-progressive cerebellar ataxia, moderate intellectual disability, and autistic behavior. At 14 years of age, a 6-month period of treatment with riboflavin (100 mg/day) was implemented. Neither clinical nor biochemical (urinary 2-HGA excretion of 318 vs. 251 mmol/mol creatinine) effect was observed. Brain MRI performed before riboflavin treatment and 6 months after cessation revealed the same leukodystrophic changes as previously, while more pronounced cortical atrophy was observed (Fig. 1).

### Molecular data

Two different heterozygous variants in the *L2HGDH* gene were identified in the patient. The first was a known substitution c.829 C > T(p.Arg277*) causing a stop gained change involving the alteration of a conserved nucleotide. It was a very rare variant in population database (was found at a frequency of 0.0000062 in 1,613,762 control chromosomes in the gnomAD database, with no homozygous occurrence). In silico tool predicted a pathogenic outcome for this variant, because this change created a premature translational stop signal (p.Arg277*) in the *L2HGDH* gene. Nonsense variant predicted to result in protein truncation or nonsense-mediated mRNA decay (NMD) in a gene for which loss-of-function (LoF) is a known mechanism of disease (Z-score = 2.339; PMIDs: 16,134,148, 20,052,767). This stop gained variant has been previously observed in the individuals affected with L-2-hydroxyglutaric aciduria (Steenweg et al. 2010; Vilarinho et al. 2005) and has been reported to the ClinVar database as Pathogenic (ID: 863,865). This variant was classified as a Pathogenic according to ACMG/AMP criteria.

The second was novel substitution c.1196 + 1G > A in canonical splice site sequence and causes a splice donor change in intron 9. It was also very rare in population database (the variant allele was found at a frequency of 0.000000686 in 1,458,258 control chromosomes in the gnomAD database, with no homozygous occurrence). It is predicted (ADA, RF, SpliceAI scores = 1) to cause abnormal gene splicing, leading to donor loss and skipping of exon 9. Abnormal transcript undergoes NMD. To date, no clinical significance assessments have been submitted for this variant to ClinVar or HGMD database. Therefore, this variant was classified as a likely pathogenic according to ACMG/AMP criteria.

The effect of both null variants is a loss of normal protein function. In *L2HGDH* gene, more than 30 pathogenic LoF variants have been reported to date (VarSome database, PMID: 37,378,753).

## Discussion

The present study described the patient with L2HGA presenting with epilepsy from 5 years of age, non-progressive cerebellar ataxia, and mild to moderate intellectual disability during follow-up. The patient presented with typical neurological manifestation of L2HGA confirmed with biochemical (increased urinary L2HGA) and molecular (including novel pathogenic variant in the *L2HGDH* gene) features.

Steenweg et al. (Steenweg et al. 2010) systematically evaluated the brain MRIs of 56 patients, and found a highly characteristic pattern of MRI abnormalities in L2HGA including abnormalities of the subcortical cerebral white matter dentate nucleus, globus pallidus, putamen, and caudate nucleus. This pattern was also observed in our patient.

The pathogenesis of L2HGA is linked with the accumulation of L2HG which is toxic to the white matter, causing myelin vacuolation. Increased L2HG in tissues induces the free radical formation and increases glutamate uptake in synaptosomes and synaptic vesicles (Rzem et al. 2004, 2007; Schaftingen et al. 2009; Junqueira et al. 2003; Latini et al. 2003). No correlation between the clinical course and the urinary excretion of L2HGA as well as no clear genotype–phenotype correlation (Bozaci et al. 2023; Sass et al. 2008; Steenweg et al. 2010; Vilarinho et al. 2005).

There have been published several case reports suggesting that therapy of flavin adenine dinucleotide (FAD) or its precursor—riboflavin with or without levocarnitine can be effective for L2HGA (Bozaci et al. 2023; Yilmaz 2009; Ahmed et al. 2023; Kular 2019; Samuraki et al. 2008; Shah et al. 2020; George et al. 2021). Table 1 provides the literature search on L2HGA cases treated with FAD/riboflavin with or without levocarnitine.

**Table 1:** Case reports of L2HGA patients treated with FAD/riboflavin with or without levocarnitine (*n.a.* non analyzed)

Patient	Treatment	Age of treatment initiation	Treatment duration	Clinical and biochemical effect	Genotype	References
1	Riboflavin 100 mg twice daily and oral L-carnitine 1500 mg twice daily	15 years and 8 months	2 months	Improvement in speech in terms of scanning quality; no changes in ataxia, tremors, or hand coordination	Homozygote; c.829 C > T, p.Arg227*	Ahmed et al. 2023
			1.5 years	Much improvement in speech, tremors, and ataxia was noted; the patient was able to lift the cup of water and drink without help; her gait was much more stable		
2	Riboflavin 100 mg twice daily and oral L-carnitine 1500 mg twice daily	18 years and 2 months	2 months	Improvement in scanning quality of speech; no change in ataxia, tremors, or hand coordination was noted	The sister of patient 1 (the same genotype)	
			1.5 years	The patient’s speech, tremors, and ataxia were much improved; she no longer needed straws to drink water		
3	Riboflavin	4 years	10 years	Progression of spasticity and epilepsy were observed	n.a	Kular 2019
4	FAD 30 mg/day and levocarnitine chloride 900 mg/day	40 years	6 months	Tremor and dystonia gradually improved; urinary L2HGA decreased by 50% after 6 months	Homozygote; c.845G > A, p.Arg282Gln	Samuraki et al. 2008
			4 years	This improvement was maintained		
6	Carnitine (dosage—not known) and riboflavin (dosage not known)	28 years	n.a	Some improvement in her tremors and gait	Homozygote; c.256 + 1 G > A (5′ splice site) at intron‐2 of the *L2HGDH* gene	Shah et al. 2020
7	Riboflavin (vitamin B2, precursor of FAD; 100 mg/day)	16 years	3 months	Partially improved cognitive and motor performance within days, and the urinary L2HGA level decreased by 75% within 3 months of treatment; cessation of riboflavin treatment resulted in significant decompensation (including clinical symptoms and urinary L2HGA excretion)	n.a	Yilmaz 2009
8	Riboflavin 60 mg/day; levocarnitine 1000 mg/day	5 years	1 year	Parents reported improvement in behavioral symptoms which was also reflected in the behavioral assessment	Homozygote; c.293 A > G, p.His98 Arg	George et al. 2021
9–19 (10 patients)	Riboflavin and levocarnitine	Mean age 53.36 ± 47.43 months (min: 6 months; max: 180 months)	n.a	Significant decrease in urinary L2HGA; neurologic improvement was seen in 3 out of 10 patients	5 different homozygous variants: c.738 + 5 A > G splice variant; c.164G > A (p.Gly55 Asp), c.528G > T (p.Glu176 Asp), c.845G > A (p.Arg282Gln), c.1115 delT (p.Met372fs)	Bozaci et al. 2023

FAD, acting as L2HG dehydrogenase (L2HGDH), may activate the oxidation of increased L2HG, which would accelerate conversion of L2HG to α‐ketoglutarate and reduce the toxicity of L2HG to the central nervous system (Rzem et al. 2007; Schaftingen et al. 2009; Bozaci et al. 2023). This hypothesis suggests that FAD acts in a chaperone function to restore L2HGDH enzyme activity and thereby reduces L2HG excretion. Levocarnitine stimulates the formation and excretion of short‐chain acylcarnitine derivatives of glutaric acid. However, FAD/riboflavin supplementation is only effective in *L2HGDH* whereas truncated enzymes (presumed null mutations) are not responsive (due to the complete absence of residual enzyme activity), which was also shown in our study (Rzem et al. 2007; Schaftingen et al. 2009; Bozaci et al. 2023).

## Conclusions

1. This study identified novel pathogenic variants in the *L2HGDH* gene in a patient with L-2-hydroxyglutaric aciduria (L2HGA). These findings contribute to the understanding of the genetic basis of L2HGA and highlight the importance of genetic testing for diagnosis and genetic counselling of affected families. Our findings on the novel variant expand the spectrum of variants known to cause L2HGA.
2. Our observation could confirm the hypothesis of riboflavin acting as a pharmacological chaperone, which was unable to augment a complete loss of normal protein (L2HGDH) function as effect of both null *L2HGDH* variants.

## Material and methods

### Genetic analysis

Genetic testing was performed in patient using genomic DNA sample automatically extracted from the peripheral blood leukocytes with a MagNA Pure (Roche Diagnostics, Rotkreuz, Switzerland) according to the manufacturer’s protocol. Next-generation sequencing (NGS) was performed with the TruSight One Sequencing Panel (Illumina, San Diego, CA, USA). Enriched libraries were paired‐end sequenced (2 × 100 bp) on the HiSeq 1500 (Illumina, San Diego, CA, USA) according to the manufacturer’s protocol. The average read depth was 90, with > 97% of the target regions covered at a depth of 20-fold. Read alignment was performed to the human genome assembly GRCh38/hg38. The detected variants were annotated using ANNOVAR and converted to MS Access format for final manual analyses. Alignments were viewed with Integrative Genomics Viewer. The annotated variants were filtered according to minor allele frequency < 0.01 in gnomAD databases (https://gnomad.broadinstitute.org) and in-house database of > 10,000 Polish individuals, conservation (Genomic Evolutionary Rate Profiling GERP), and predicted impact on protein structure and function. Eleven different in silico prediction tools were used for the interpretation of candidate missense variants, including REVEL initially and additional AlphaMissense, CADD, EIGEN, FATHMM-MKL, MutationTaster, PolyPhen-2, and SIFT. Variants occurring at splice sites were initially analyzed using SpliceAI, with additional evaluation supported by ADA, MaxEntScan, and RF to assess their potential impact on normal splicing. Finally the candidate variants were classified according to the American College of Medical Genetics and Genomics and the Association for Molecular Pathology (ACMG/AMP) guidelines. According to the criteria, benign and likely benign variants were filtered out, thereby retaining only pathogenic, likely pathogenic changes, and variants of uncertain significance (VUS). The nomenclature of molecular variants follows the Human Genome Variation Society guidelines (HGVS, http://varnomen.hgvs.org/) using MANE select human L2HGDH reference sequence: NM_024884.3 (for cDNA) and NP_079160.1 (for protein). The candidate pathogenic variants were verified in the proband and their parents by Sanger sequencing using BigDye Chemistry and 3130 Genetic Analyzer (Applied Biosystems).

Written informed consent for genetic studies performed in the patient was obtained from the parents. This study has been carried out in accordance with the declaration of Helsinki for experiments involving humans.

5-year-old girl. First row. Increased signal intensity of both hilum of dentate nuclei. Symmetrically increased volume of caudate and lenticular nuclei with increased signal intensity, especially of the both globi pallidi. Abnormally increased signal of subcortical white matter in the cerebral hemispheres with predominance of frontal lobes. No signs of diffusion restriction and no contrast enhancement, no signs of hemorrhage/calcifications in the SWI sequence (not shown). Follow-up examination performed 1 year later. Second row. The location of previously described changes has not changed. Basal ganglia signal intensity is slightly lower, while higher signal is visible in the subcortical areas of white matter. The sulci between the convolutions of the cerebral cortex have slightly widened supratentorially. MRI scan performed at the age of 14 and 15. Third and fourth rows. The supratentorial ventricular system dilated atrophically. Cortical atrophy is visible. The volume of the globi pallidi has decreased. The increased signal intensity of the white matter in the frontal lobes occupies slightly larger areas.

## Data Availability

All data generated or analysed during this study are included in this published article.
